# Supplementary material for: Coumarin Ketoxime Ester with Electron-Donating Substituents as Photoinitiators and Photosensitizers for Photopolymerization upon UV-Vis LED Irradiation
Source: Polymers (Basel). 2022 Oct 28;14(21):4588. doi: 10.3390/polym14214588 (PMC9655686; doi:10.3390/polym14214588)
Supplement: Supplementary file 1 [file polymers-14-04588-s001.zip › polymers-1991405-supplementary.pdf]

Supporting information

Coumarin Ketoxime Ester with Electron-donating Substituents as Photoinitiators and Photosensitizers for Photopolymeriza-tion upon UV–Vis LED Irradiation

^{1}H and ^{13}C NMR spectrum of the compounds :

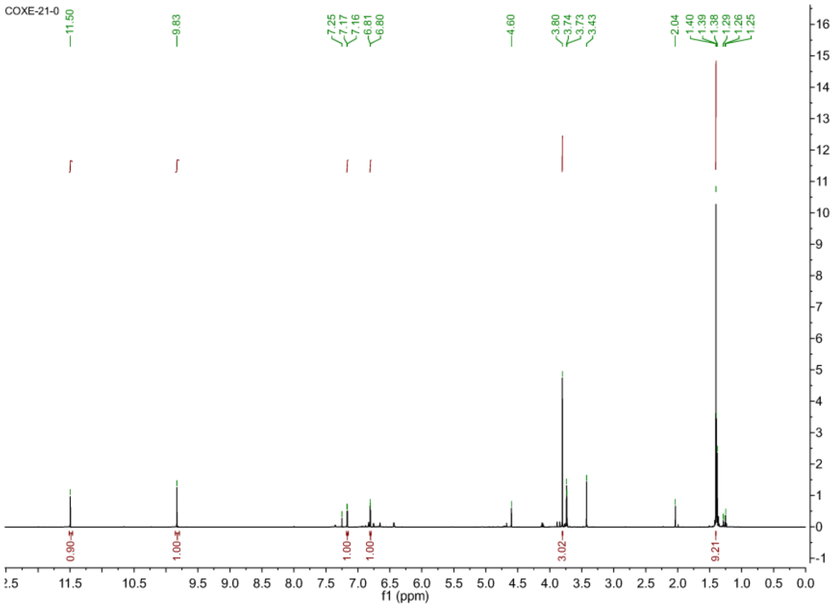

Figure S1. ^{1}H NMR spectrum of 3-(1,1-dimethylethyl)-2-hydroxy-5-methoxy- Benzaldehyde (Salicylaldehyde)

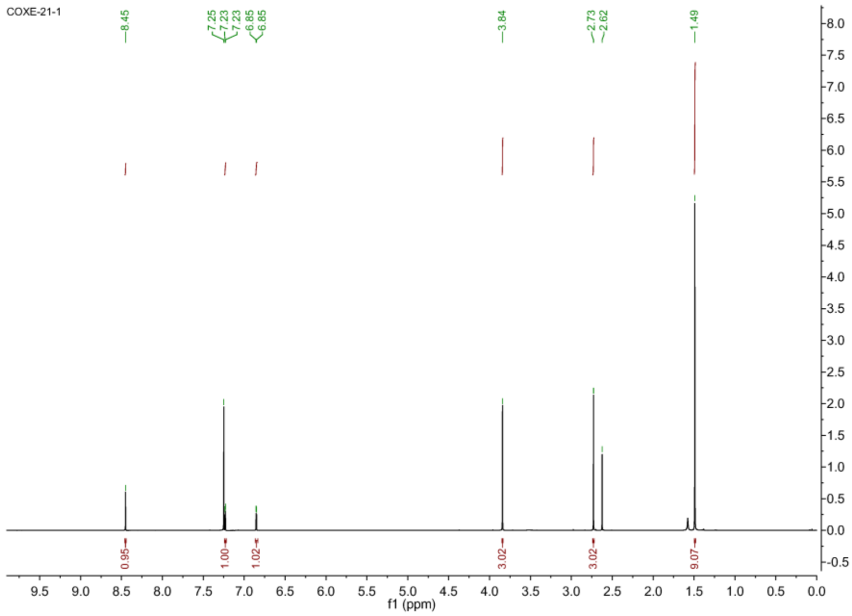

Figure S2. ^{1}H NMR spectrum of 3-acetyl-8-(1,1-dimethylethyl)-6-methoxy-2H-1-Benzopyran-2-one (Coumarin-ketone).

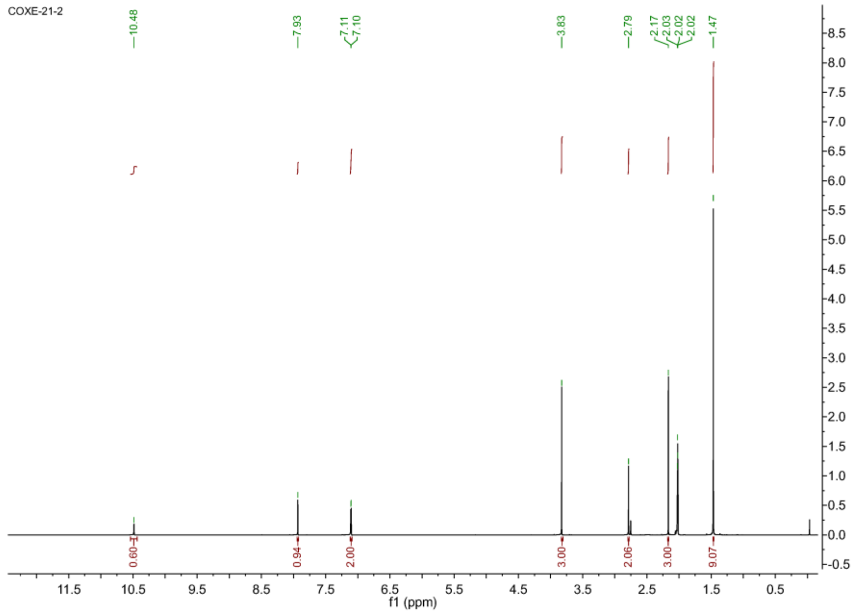

Figure S3. ^{1}H NMR spectrum of 3-[1-(hydroxyimino) ethyl]-8-(1,1-dimethylethyl)-6-methoxy-2H-1-Benzopyran-2-one (Coumarin-ketoxime).

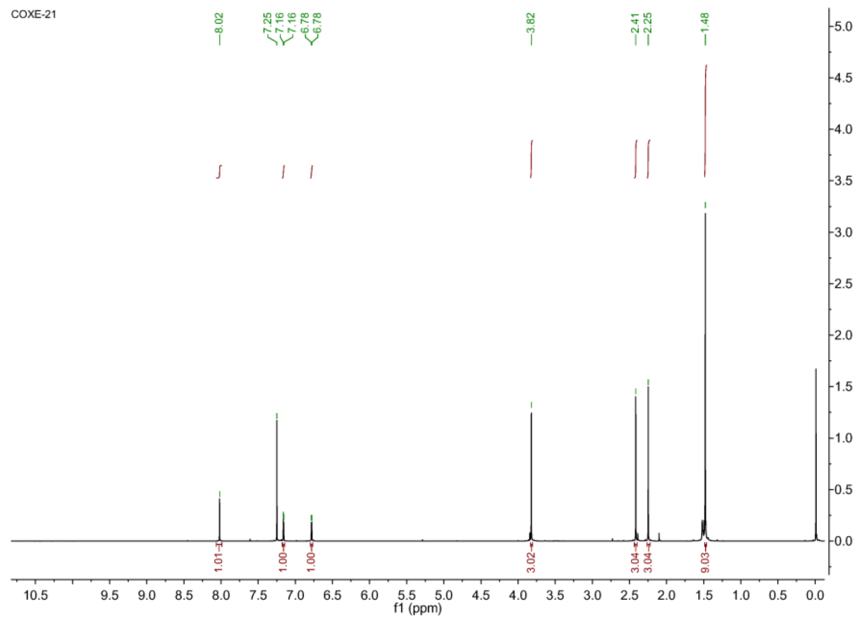

Figure S4. ^{1}H NMR spectrum of 3-[(1-[(acetyloxy) imino] ethyl]-8-(1,1-dimethylethyl)-6-methoxy-2H-1-Benzopyran-2-one (Coumarin-ketoxime ester, COXE-O).

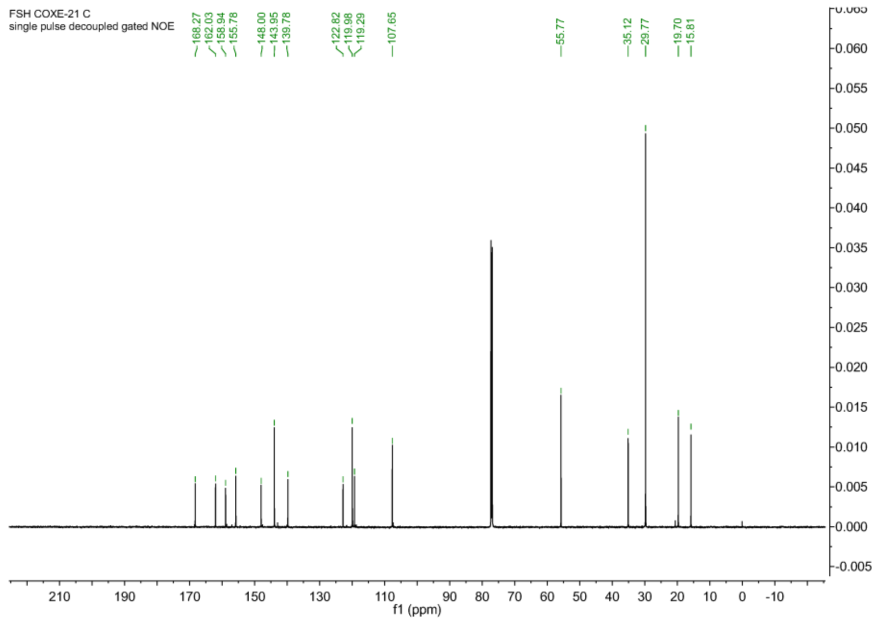

Figure S5. ^{13}C NMR spectrum of 3-[(1-[(acetyloxy) imino] ethyl]-8-(1,1-dimethylethyl)-6-methoxy-2H-1-Benzopyran-2-one (Coumarin-ketoxime ester, COXE-O).

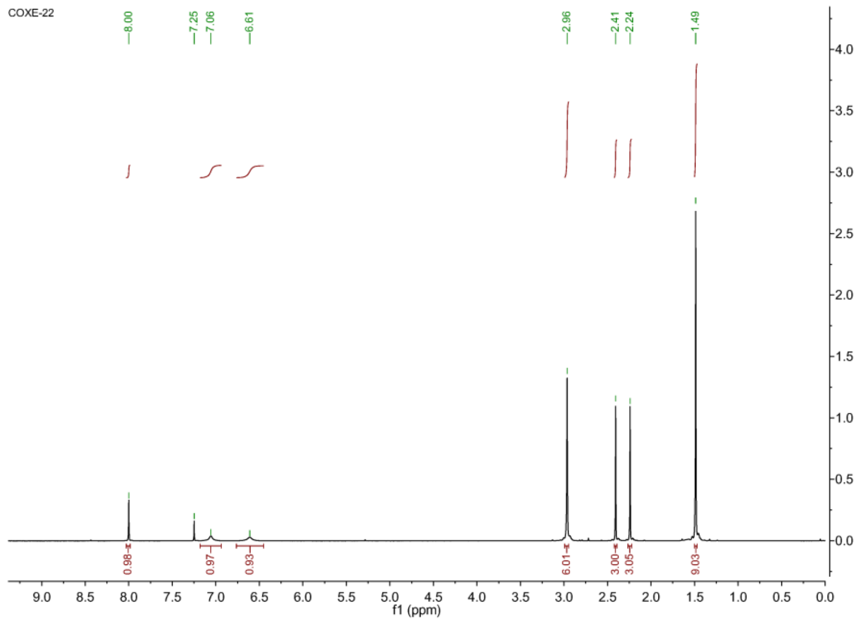

Figure S6. ^{1}H NMR spectrum of 3-[(1-[(acetyloxy) imino] ethyl]-8-(1,1-dimethylethyl)-6-dimethylamino-2H-1-Benzopyran-2-one (COXE-N).

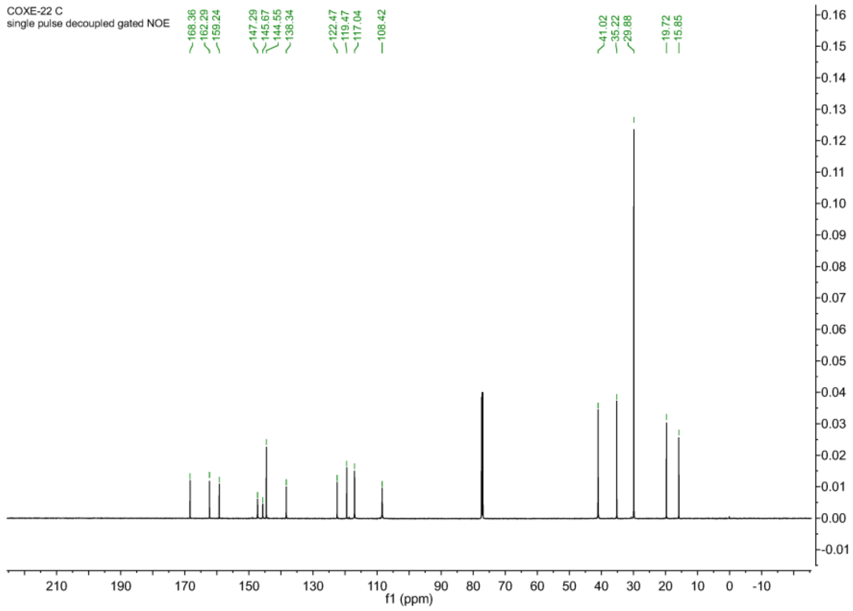

Figure S7. ^{13}C NMR spectrum of 3-[(1-[(acetyloxy) imino] ethyl]-8-(1,1-dimethylethyl)-6-dimethylamino-2H-1-Benzopyran-2-one (COXE-N).

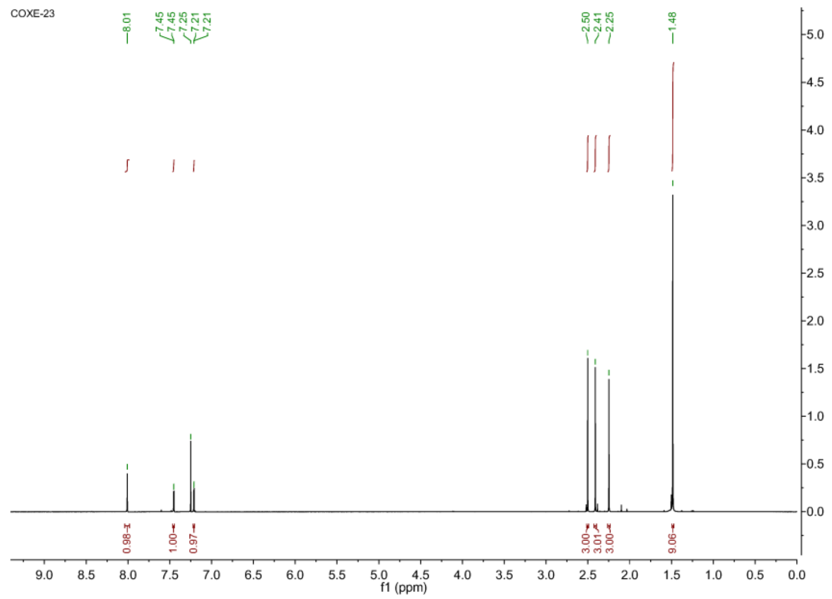

Figure S8. ^{1}H NMR spectrum of 3-[(1-[(acetyloxy) imino] ethyl]-8-(1,1-dimethylethyl)-6-dmethylthio-2H-1-Benzopyran-2-one (COXE-S).

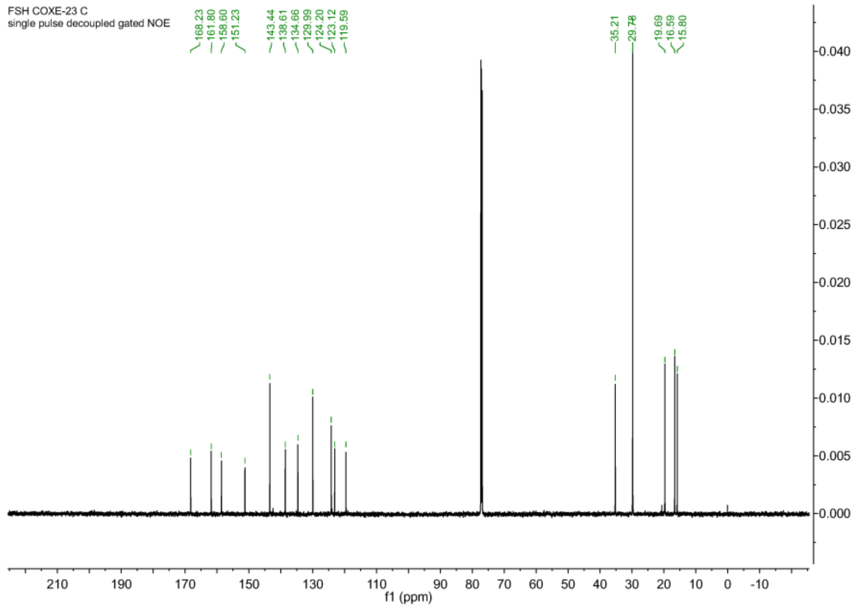

Figure S9. ^{13}C NMR spectrum of 3-[(1-[(acetyloxy) imino] ethyl]-8-(1,1-dimethylethyl)-6-dmethylthio-2H-1-Benzopyran-2-one (COXE-S).

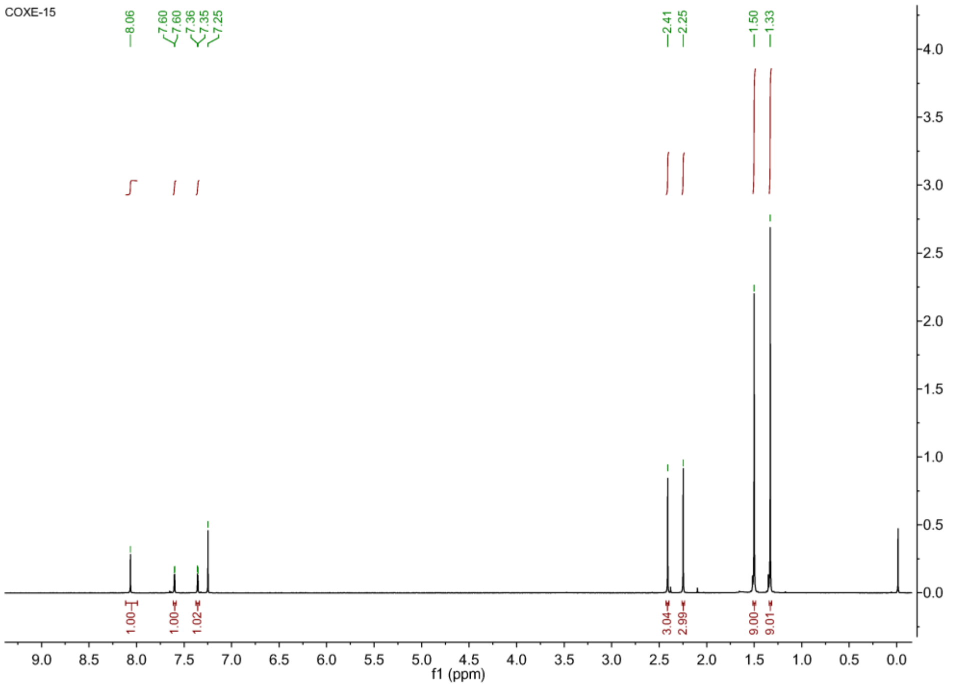

Figure S10. ^{1}H NMR spectrum of 3-[(1-[(acetyloxy) imino] ethyl]-6,8-bis(1,1-dimethylethyl)-2H-1-Benzopyran-2-one (COXE-C)

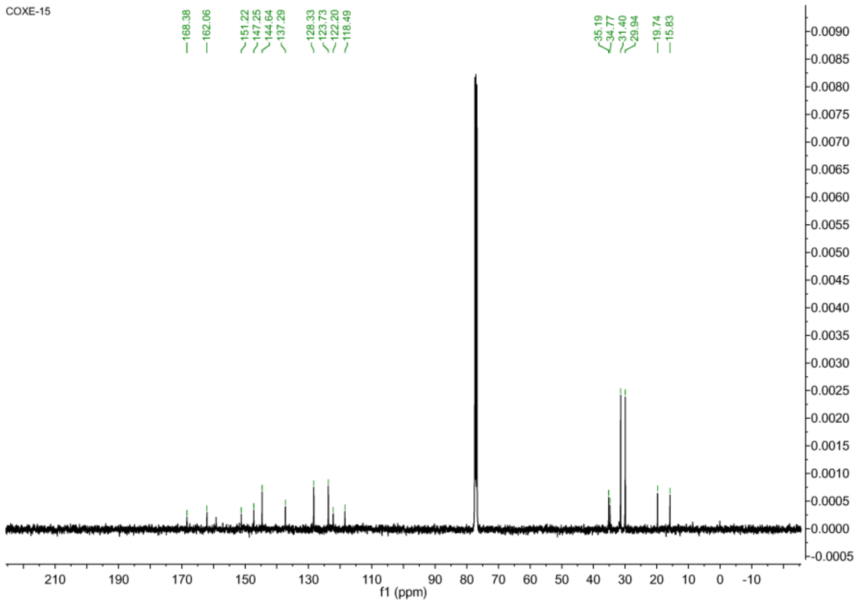

Figure S11. ^{13}C NMR spectrum of 3-[(1-[(acetyloxy) imino] ethyl]-6,8-bis(1,1-dimethylethyl)-2H-1-Benzopyran-2-one (COXE-C)

Table S1. Molar extinction coefficients (ε, $M^{-1}\cdot cm^{-1}$) of COXEs in acetonitrile at common LED emission wavelengths

	**ε (M^{-1} cm^{-1})**					
	**365nm**	**385nm**	**400nm**	**425nm**	**450nm**	**475nm**
COXE-C	3.03×10^{3}	6.58×10^{2}	1.0×10^{2}	0	0	0
COXE-O	5.42×10^{3}	3.02×10^{3}	1.10×10^{3}	1.44×10^{2}	40	0
COXE-N	9.63×10^{2}	1.63×10^{3}	2.06×10^{3}	2.18×10^{3}	1.49×10^{3}	6.54×10^{2}

COXE-S	3.96×10^3	3.38×10^3	2.08×10^3	4.17×10^2	2.0×10^2	20

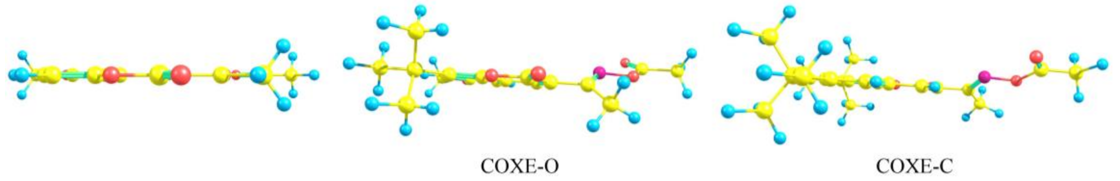

Figure S12. Geometrically optimized horizontal structures of COXE using DFT calculations at B3LYP/6-31G(d) level.

LC-MS spectrum of the photoproducts

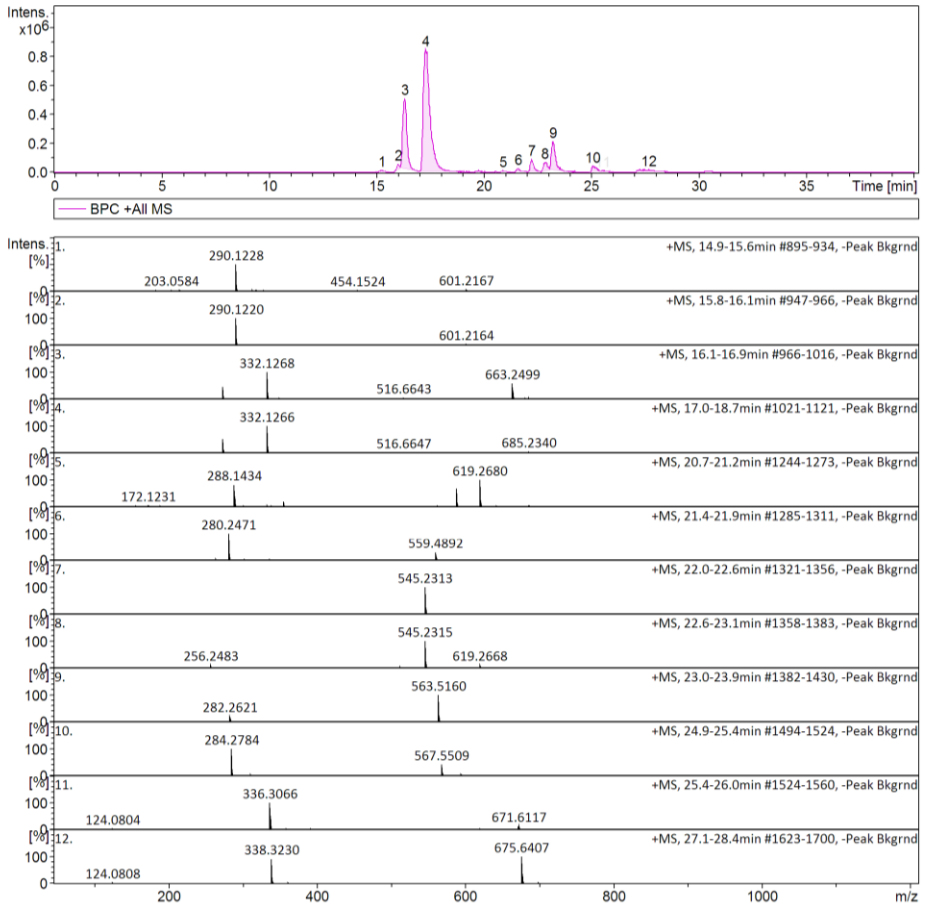

Figure S13. LC-Signals and Mass spectrum of the photoproducts of COXE-O after exposure 30min via LC-MS measurement (light source: 400nm LED, light intensity: 100mW·cm^{-2}).

Photodecarboxylation Experiment

From the photodecarboxylation experiment in Figure S13, it indicates that using bromocresol green as pH indicator (bottle on the right, dissolved in water) to detect carbon dioxide generated by COXE-C (bottle on the right, dissolved in acetonitrile). When COXE-C is continuously irradiated by 365 nm LED after 30min, the color of indicator aqueous solution changes from blue (neutral) to yellow green (weak acidity), which proves the formation of carbon dioxide and further proves that the decarboxylation reaction of acetoxy radical occurs.

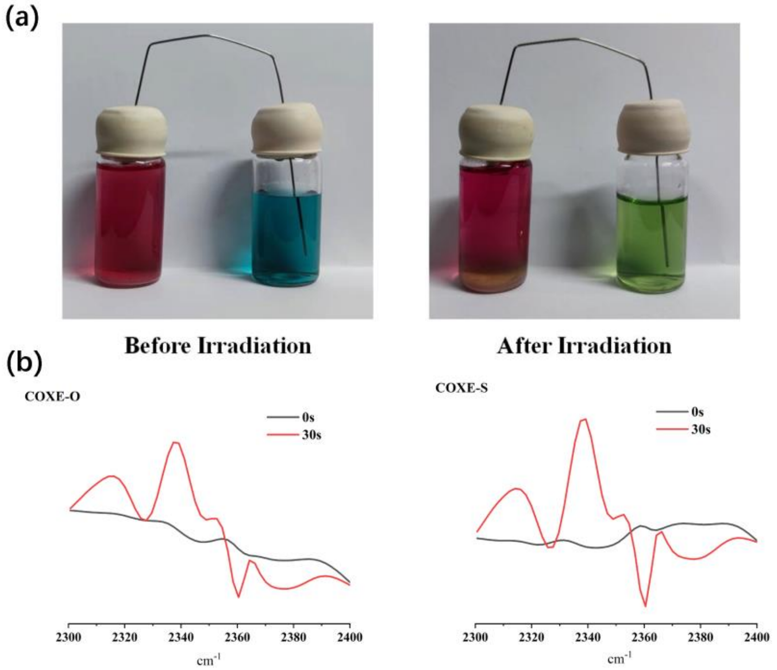

Figure S14. (a) Photodecarboxylation experiment of irradiation at 365 nm LEDs for COXE-C dissolved in acetonitrile (right side) on the condition of bromocresol green dissolved in water (left side). (b) The CO_2 detection of IR spectrum of COXE-O and COXE-S upon 400nm LED irradiation in 0s and 30s.

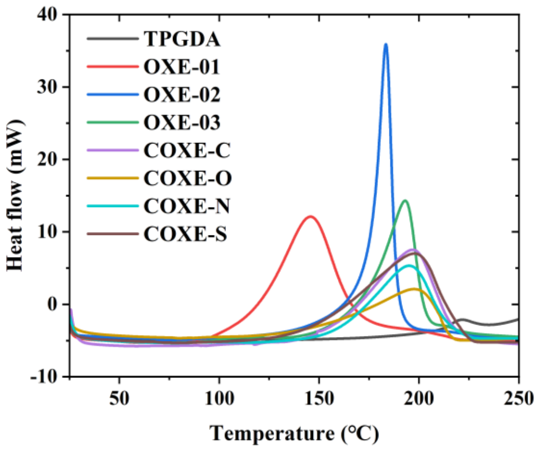

Figure S15. DSC measurements of COXEs in TPGDA (COXEs concentration: 4.8×10^{-5} mol·g^{-1}).

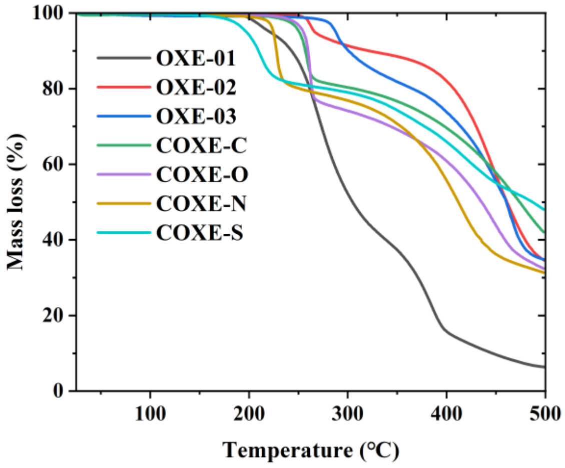

Figure S16. TGA of COXEs

Table S2. Initial thermal decomposition temperature of COXEs and commercial oxime ester PIs OXE-01, OXE-02 and OXE-03 measured by TGA.

	OXE-01	OXE-02	OXE-03	COXE-C	COXE-O	COXE-N	COXE-S
Decomposition temperature(℃)	201	257	278	241	246	219	174

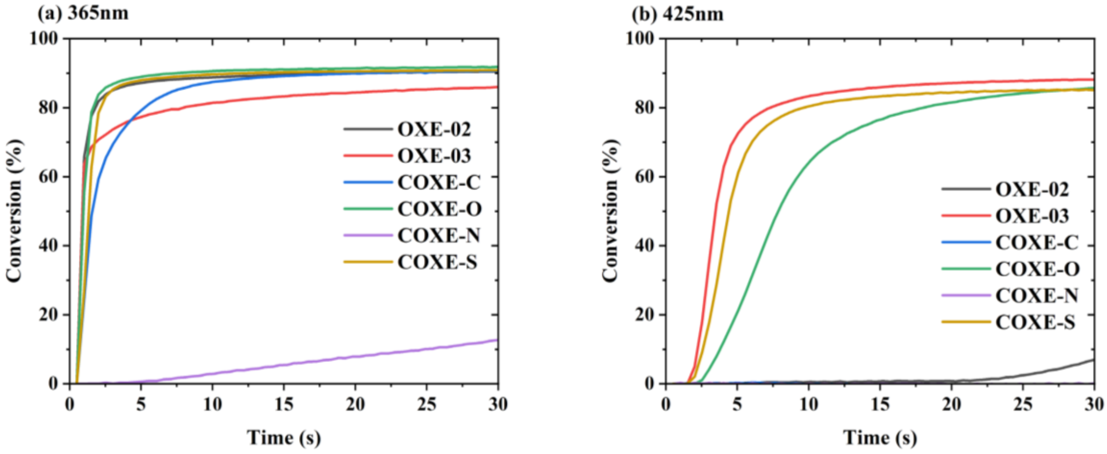

Figure S17. C=C double bond conversion curves of TPGDA polymerization initiated by COXEs, OXE-02 and OXE-03 (4.8×10^{-5} mol·g^{-1}) under LED irradiation at (**a**) 365 nm and (**b**) 425nm LED in 30s (LED light intensity: 100mW·cm^{-2}).

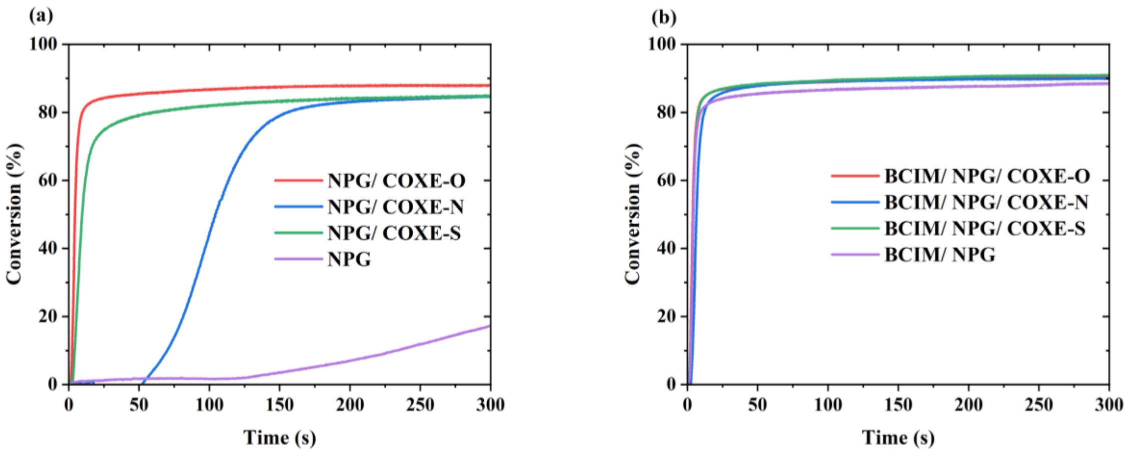

Figure S18. C=C double bond conversion-time curves of TPGDA polymerization initiated by (**a**) NPG/ COXEs, (**b**) BCIM/ NPG/ COXEs under 400nm LED irradiation (light intensity: 100mW·cm^{-2}). The concentration of COXEs: 2.4×10^{-6} mol·g^{-1}, the concentration of NPG and BCIM: 7.2×10^{-6} mol·g^{-1}.

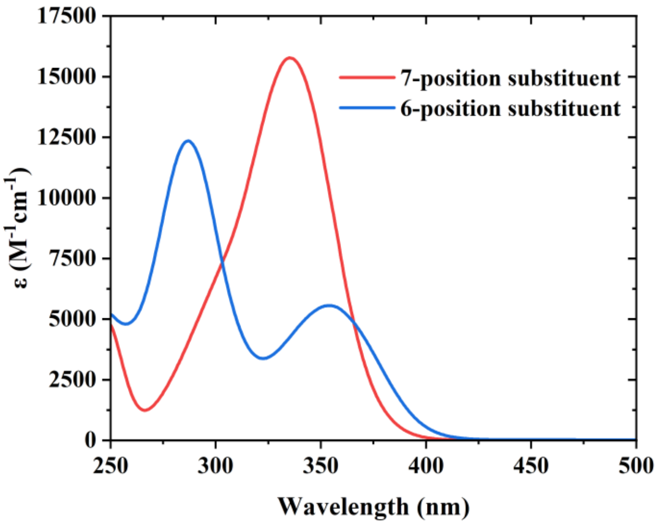

Figure S19. UV−Vis absorption spectra and for 6-position and 7-position methoxy substituted coumarin oxime esters in acetonitrile at room temperature.

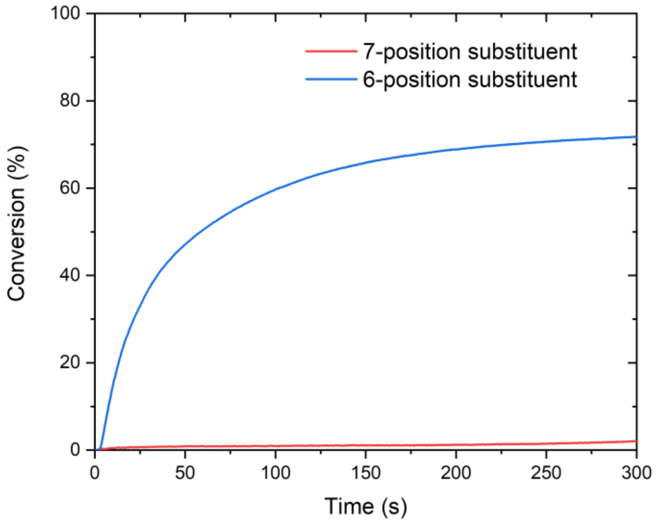

Figure S20. C=C double bond conversion-time curves of TPGDA polymerization initiated by 6-position and 7-position methoxy substituted coumarin oxime esters under 400nm LED irradiation (light intensity: 100mW·cm^{-2}). The concentration of coumarin oxime esters: 2.4×10^{-6} mol·g^{-1}.
